# Comparing multiple infection control measures in a nursing home setting: a simulation study

**DOI:** 10.1017/ice.2024.43

**Published:** 2024-07

**Authors:** Haomin Li, Daniel K. Sewell, Ted Herman, Sriram V. Pemmeraju, Alberto M. Segre, Aaron C. Miller, Philip M. Polgreen, for the CDC MInD-Healthcare Group

**Affiliations:** 1 Department of Biostatistics, University of Iowa, Iowa City, IA, USA; 2 Department of Computer Science, University of Iowa, Iowa City, IA, USA; 3 Department of Internal Medicine, University of Iowa, Iowa City, IA, USA; 4 Departments of Epidemiology, University of Iowa, Iowa City, IA, USA

## Abstract

**Objective:**

Compare the effectiveness of multiple mitigation measures designed to protect nursing home residents from infectious disease outbreaks.

**Design:**

Agent-based simulation study.

**Setting:**

Simulation environment of a small nursing home.

**Methods:**

We collected temporally detailed and spatially fine-grained location information from nursing home healthcare workers (HCWs) using sensor motes. We used these data to power an agent-based simulation of a COVID-19 outbreak using realistic time-varying estimates of infectivity and diagnostic sensitivity. Under varying community prevalence and transmissibility, we compared the mitigating effects of (i) regular screening and isolation, (ii) inter-resident contact restrictions, (iii) reduced HCW presenteeism, and (iv) modified HCW scheduling.

**Results:**

Across all configurations tested, screening every other day and isolating positive cases decreased the attack rate by an average of 27% to 0.501 on average, while contact restrictions decreased the attack rate by an average of 35%, resulting in an attack rate of only 0.240, approximately half that of screening/isolation. Combining both interventions impressively produced an attack rate of only 0.029. Halving the observed presenteeism rate led to an 18% decrease in the attack rate, but if combined with screening every 6 days, the effect of reducing presenteeism was negligible. Altering work schedules had negligible effects on the attack rate.

**Conclusions:**

Universal contact restrictions are highly effective for protecting vulnerable nursing home residents, yet adversely affect physical and mental health. In high transmission and/or high community prevalence situations, restricting inter-resident contact to groups of 4 was effective and made highly effective when paired with weekly testing.

## Introduction

Residents of nursing homes and long-term care facilities are at high risk of being severely impacted by infectious disease outbreaks due to both age and comorbidities.^1^ Additionally, the relatively dense connections among both residents and healthcare workers (HCWs) can lead to unusually rapid diffusion of infectious pathogens. This has been evident throughout the COVID-19 pandemic, where there have been over 1.6 million cases and 166 thousand deaths among US nursing home residents, as well as over 2,000 deaths among staff/HCWs.^2^ Residents’ mortality rate has been over 100 times greater than that of the general population and over 20 times greater than that of the population over 65 years of age.^3^

Current documented strategies aimed at mitigating the risk of COVID-19 to nursing home residents include isolation and contact restrictions, personal protective and hygienic measures, health education and information sharing, monitoring, screening, and entry regulation measures.^4,5^ However, direct evidence connecting such interventions to outcomes is lacking, so high-quality modeling studies are necessary to provide more confidence in the effects of various mitigation measures.^5,6^

Unlike observational studies, modeling studies have the advantage of avoiding logistical and ethical hurdles when evaluating various strategies and comparing counterfactual scenarios. For example, the impact of varying surveillance strategies has received much attention,^6–9^ and this is also true in modeling studies.^10–14^ Rarely have surveillance and isolation been compared against other measures in a nursing home or long-term care facility setting, with the exception of vaccination^11^ and at least one form contact restriction based on disease status/history^10^. These studies’ results varied widely, from very effective when the diagnostic test sensitivity is very high^11,13^ to potentially counterproductive in other settings such as incarceration facilities with highly seasonal disease incidence.^14^

The goal of this work is to evaluate and compare the efficacy of strategies including (i) screening and isolation, (ii) structured resident contact restrictions, (iii) reduced HCW presenteeism, and (iv) modified HCW scheduling over a wide variety of community prevalence and disease transmissibility parameters. Toward this aim, we used spatially and temporally fine-grained HCW location data obtained from sensor motes in a small nursing home setting to support an agent-based model (ABM) that compares various counterfactual scenarios related to the spread of COVID-19 like respiratory disease.

## Methods

### Overview

We deployed a sensor mote system of our own design in a 32-room residential care facility in July of 2019. The system consists of rechargeable *badges* worn by HCWs and line-powered *beacons* placed in fixed locations to act as geographical markers and data aggregators. Badges periodically broadcast an identifying packet; when a beacon detects such a message, it logs (i) the sender’s id, (ii) the received signal strength index (an observable proxy for physical distance), and (iii) the time the packet was received. Fifteen beacons were placed in hallways, 4 at the nursing station, 2 in the two dining/commons, and 32 in residents’ rooms (all rooms are single occupancy). By fusing beacon data, we can pinpoint when a badge and its associated HCW entered/exited a room.

Because the 32 residents had limited mobility, we were able to reliably infer their location (either their assigned room or one of the common areas) by observing HCW behavior; for example, the data clearly show when HCWs escort residents to the dining rooms for meals or back to their rooms after dinner. For this study, we leveraged 8 hours of data (7:00–15:00) collected from the badges of the 17 HCWs working on July 17, 2019. The data were partially replicated to recreate a full day’s HCW/resident interactions in a manner that reflects the facility’s dining and activity schedule when staffed by 34 HCWs working in shifts.

### Contact networks

Using the timestamps and locations of each HCW or resident, we constructed an *empirical contact network* consisting of nodes representing agents (HCWs or residents) and weighted edges representing the cumulative time the agents spent in the same location for the day. Three alternate contact networks were also constructed from the empirical contact network in a manner consistent with a specified type of infection control intervention:

1. (*Lockdown network*) No resident-resident interactions were allowed, that is, all the residents remain in their assigned rooms throughout the day.
2. (*Homogeneous network*) Every resident is relocated to a single common area for a prespecified number of social interaction hours each day: pertinent resident/HCW interactions are also remapped accordingly.
3. (*Bubble network*) Each resident is randomly assigned to one of N equal sized social “bubbles.” Each bubble is assigned to a distinct common area for a prespecified number of social interaction hours each day: pertinent resident/HCW interactions are also remapped accordingly (see Figure 1 for an example). Note that the homogeneous network is itself a bubble network with *N* = 1.
  **Figure 1.**
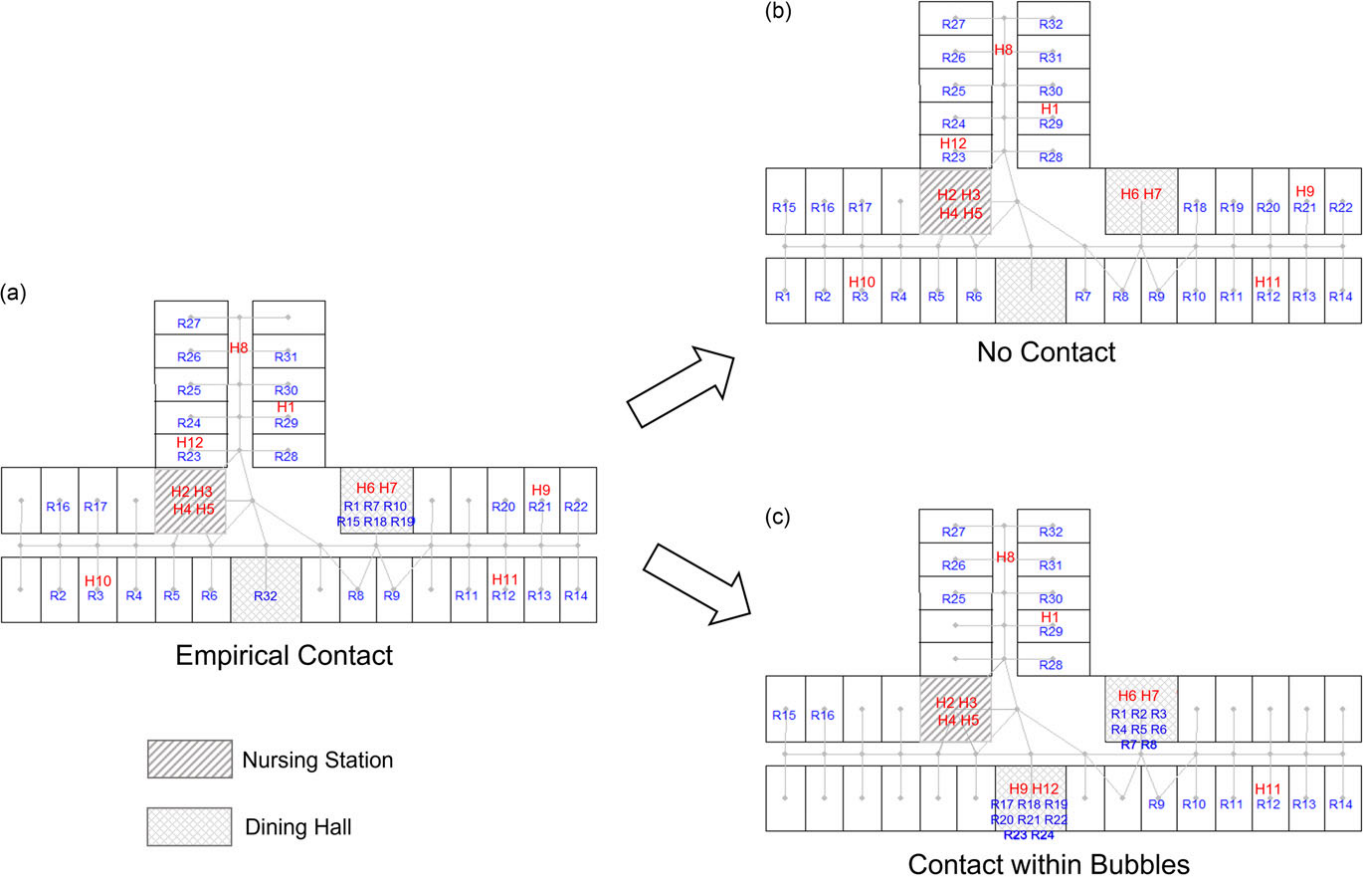
Illustration of how contact networks were constructed from sensor mote data. (a) Empirical contact network based on HCW and inferred resident locations at each time point. (b) No contact between residents was imposed, keeping all residents in their own room (corresponding to lockdown network). (c) Contact between residents was constrained to be between those assigned to the same bubble; residents within same bubbles went to dining hall at same time, as did the HCWs who were in their rooms in empirical network. The bubble network was generated based on this. *Note:* HCW, healthcare worker.

### Simulation

Each of the above contact networks can be used as the basis for an ABM and corresponding simulations. In our simulations, each individual agent (HCW or resident) will be denoted as *susceptible*, *infected*, or *recovered*.^15^ Once the parameters of a simulation are established, a randomly selected on-duty HCW is infected and all other agents are susceptible. We then perform a series of operations on the selected contact network 50 times, once for each simulated day, changing each agent’s status as the simulation demands. Infected agents infect susceptible agents according to a prespecified *transmission model*. Once infected, some agents will exhibit symptoms, while others will remain asymptomatic: both will progress through a prespecified *infection model* where the transmissibility varies over time, until they progress to the recovered state after a random-length interval. Because our simulations are short, we do not allow recovered agents to be reinfected. At the end of the simulation, we compare the *resident attack rates* (percentage of resident agents infected over the course of the simulation) to assess differences between simulation conditions.

For each simulated day:

1. (*Permute the contact network*) Randomly permute the mapping of HCW identities to HCW nodes; while the pattern of interactions is replayed each day, the HCWs replaying each role changes randomly on a daily basis, thereby ensuring that HCWs were not constrained to interact with the same residents each day.

2. (*Reallocate work*) For each resident–HCW contact involving an isolated HCW (see steps 3 and 4), reassign the contact to the HCW with the lowest workload among those available at the time of contact.

3. (*Testing and mandatory isolation*) Selected HCWs and residents are tested on a fixed prespecified schedule, where we assume the test has perfect specificity, but its sensitivity depends on time since infection. Any agent with a positive result is mandatorily isolated for 10 days until recovered according to the infection model.

4. (*Self-isolation*) Non-isolated infected agents who become symptomatic may self-isolate with a certain probability.

5. (*Internal transmission*) Susceptible agents may contract the disease during contact with a non-isolated infected agent according to the contact network edge weight (duration of contact) and how long the infected agent has been infected.

6. (*Community transmission*) Non-isolated susceptible HCWs have a certain probability of becoming infected through external sources based on the *community risk* value.

7. (*External transmission*) Each non-isolated resident will have 2 hours of interactions with two visitors (total 4 hours) each week; visitors may be infected, again in accordance with the *community risk* value, and are not tested prior to visiting.

### Model parameters and counterfactuals

The ABM parameters were divided into two groups: disease parameters derived from the existing literature and intervention parameters (Table 1). While the goal of our study was not exclusively focused on COVID-19, we used this as our model disease due to the large body of literature estimating the required parameter values.

**Table 1.** Agent-based model parameters

Disease parameters	
Incubation period^21^	Log-normal (1.54, 0.47^2)
Probability of being asymptomatic^18^	0.6
Transmission ability ( 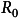 )^18,^ ^19^	3.15 (wild type)
	5.94 (delta variant)
Community risk	0.01, 0.025, 0.05, 0.1
Infectiousness profile^17^	Gamma (17, 1.3) with location shift of 13
**Intervention parameters**	
Contact profile	No contact
	Homogeneous contact
	Residents contacting each other within four bubbles
	Residents contacting each other within eight bubbles
	Unrestricted contact
Screening option	Screening every 6 days
	Screening every other day
	No screening
Sensitivity profile^16^	See main text
Residents bubble contact time per day	0.5 h, 1 h, 1.5 h, 2 h
Probability of self-isolation for HCWs with COVID-19	0
	0.18
	0.59
	0.795
	1

To obtain a realistic temporally varying *test sensitivity* curve, we estimated the severe acute respiratory coronavirus virus 2 (SARS-CoV-2) polymerase chain reaction (PCR) test sensitivity as a function of time since infection by fitting a non-linear logistic regression using natural cubic splines to data collated by Kucirka et al.^16^

The *community risk*, that is, community disease prevalence, was fixed at either 0.01, 0.025, 0.05, or 0.1. HCWs are randomly infected from external sources at an incidence rate derived from dividing the community prevalence by the number of days having transmission rate larger than 0.01 (14 days). The probability of visitors being infected is proportional to the prevalence, and the infectiousness is determined by randomly selecting the number of days since becoming infected.

The probability that an infectious individual infected a susceptible contact was derived from assuming an exponential distribution on the time to transmission with rate proportional to the shedding profile in the literature^17^ and the time in contact. Qualitatively, this led to a transmission rate very small up until a week prior to symptom onset, where it rose to peak at the day prior to symptom onset and then began to steadily decline. The proportionality constant was derived by finding the root of the equation 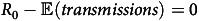 . The basic reproduction number 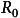 was set to be either 3.15, corresponding to the wild strain of COVID-19,^18^ or 5.94, corresponding to the delta strain.^19^ Moreover, we also assumed that residents and HCWs would be wearing masks, reducing the 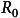 of the general population by 66%.^20^ Upon becoming infected, an agent is given an incubation period generated from the distribution in the literature, followed by an additional 2 weeks until becoming recovered.^21^

We analyzed the following three testing strategies: all residents and HCWs on the working shift are scheduled for PCR tests (1) every 6 days, (2) every other day, and (3) no testing. We tested all three strategies under restricted contacts within bubbles considering three bubble sizes (*N* = 1, 4, or 8) and four contact times (*t* = 0.5, 1, 1.5, or 2 hours/day).

We simulated varying levels of presenteeism among HCWs by considering five self-isolation probabilities (0, 0.180, 0.590, 0.795, and 1) chosen based on observed presenteeism rate of 0.82 among HCWs,^22^ decreasing presenteeism by both ½ and ¼, as well as the extremes of always and never going to work ill.

Work shift arrangements were also evaluated. Assuming two HCW shifts, each shift worked a specific number of consecutive days ranging from 1 to 14 followed by that same number of days off. For example, shift 1 may work 7 days and take 7 days off.

Unless stated otherwise, in each configuration described above, we picked as default settings residents’ contacts restricted to 4 bubbles for 2 hours each day in our simulations, community risk = 0.025, 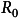 = 3.15, HCWs rotated shifts every 6 days, testing every 6 days, and 0.18 self-isolation probability after becoming symptomatic. All total, there were 208 scenarios, and for each, we ran 2,000 simulations.

## Results

The results from the nursing home ABM are reported in terms of the attack rate for nursing home residents, that is, the average proportion of the 32 residents infected within the 50-day simulation window.

**Figure 2.**
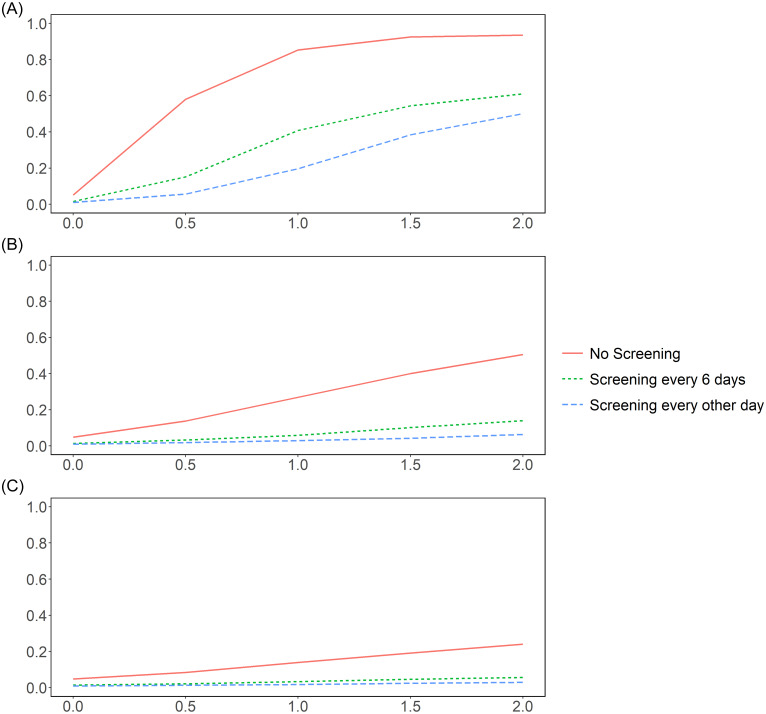
The attack rates for residents by different screening strategy. The X axis is the total hours of contact between residents; Y axis is the attack rates. (A) Residents contact each other homogeneously. (B) Residents contact each other within four bubbles. (C) Residents contact each other within eight bubbles.

Figure 2 depicts the effect of different testing strategies. For all three resident contact profiles (homogeneous (A), four bubbles (B), and eight bubbles (C)), the attack rate decreased by 0.32, 0.37, and 0.18, respectively, when going from no testing to testing every 6 days. However, there were much smaller additional reductions in attack rates of 0.11, 0.076, and 0.027, respectively, when moving from testing every 6 days to testing every other day.

**Figure 3.**
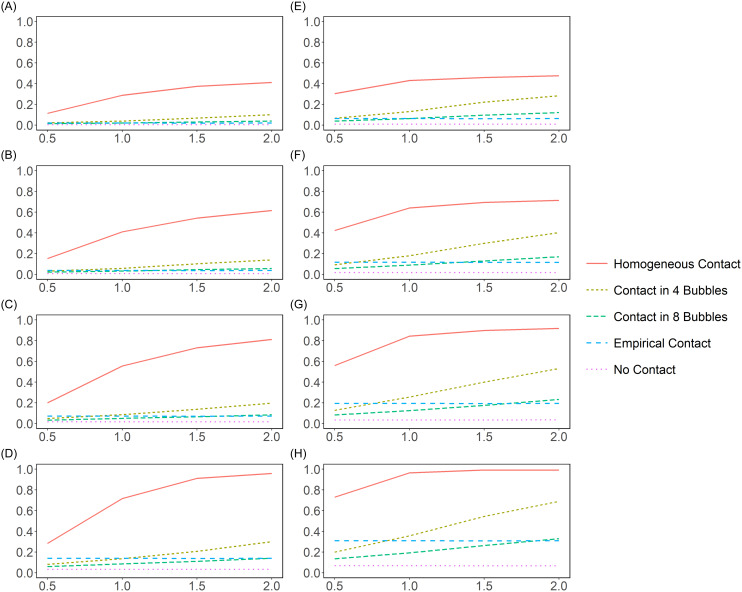
The attack rates in nursing home residents by different resident-resident contact profiles. The X axis is the total hours of daily contact between residents; the Y axis is the attack rates among residents. (A–D) R_0 of 3.15. (E–H) R_0 of 5.94. (A, E) A community prevalence of 0.01. (B, F) A prevalence of 0.025. (C, G) A prevalence of 0.05. (D, H) A prevalence of 0.1.

Figure 3 shows the effect of changing the contact profile on the mean attack rate across different 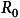 (columns) and community prevalence values (rows). While the attack rate reduction from homogeneous contact to the empirical contact patterns was generally dramatic (up to 0.82), the difference between 4 bubbles of 8 residents each and 8 bubbles of 4 residents each depended on the amount of time residents spent with their bubble; for 0.5 hours/day, the reduction in attack rates from 8 to 2 bubbles ranged across our simulation settings from 0.01 to 0.07, while ranging from 0.06 up to 0.36 for 2 hours/day.

**Figure 4.**
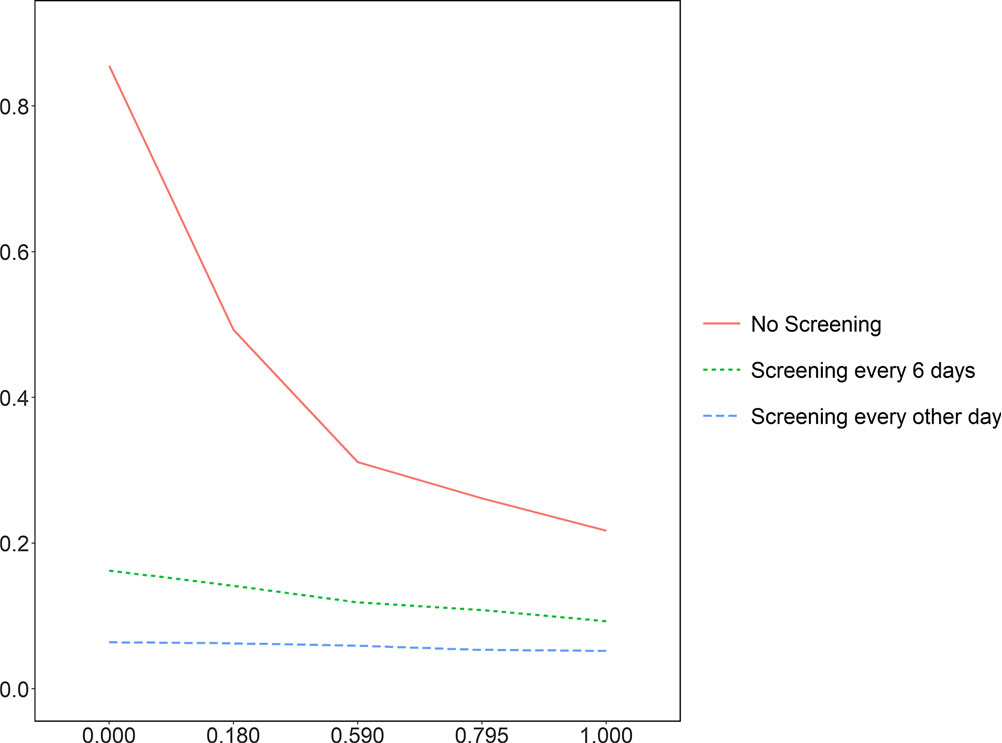
The attack rates for residents by different willingness to self-isolate. The X axis is the probability of self-isolation after an individual develops COVID-19 symptoms; Y axis is the attack rates.

Figure 4 shows the effect of self-isolation on the attack rate. In the absence of screening, changing the self-isolation probability from 0 to 1 greatly reduced the attack rate (reduction of 0.64), while this only minimally reduced the attack rate when screening every 6 days and every other day (reductions of 0.07 and 0.01 respectively). These results match expectations, as screening leads to HCWs who would otherwise engage in presenteeism undergoing mandatory isolation.

**Figure 5.**
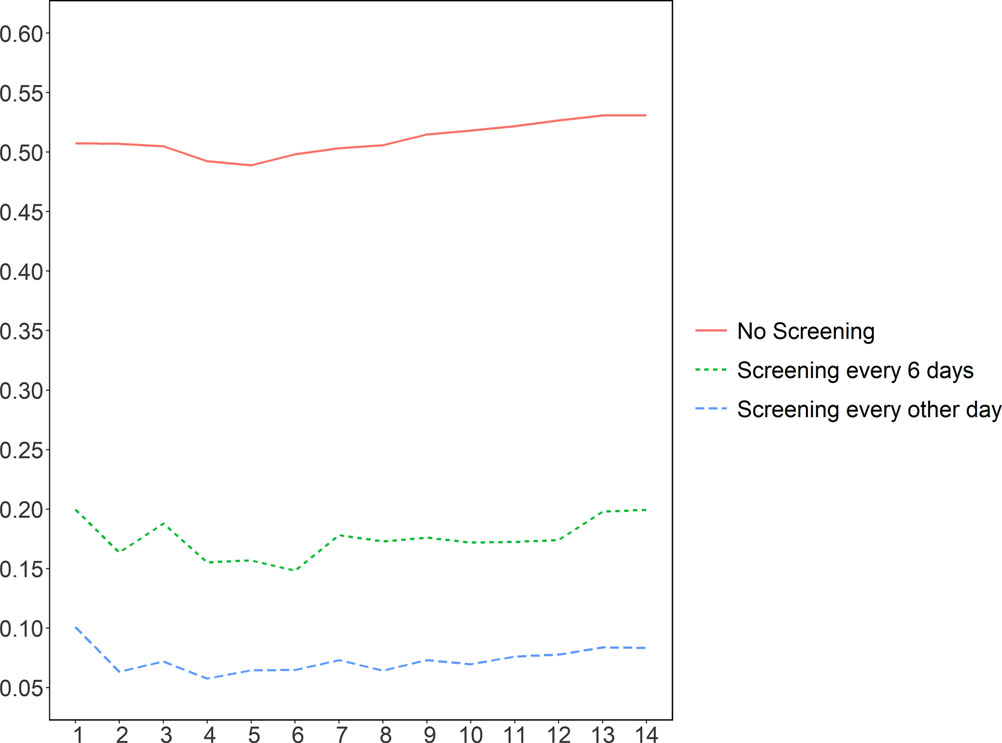
The attack rates for residents by different working shift length (ie, number of consecutive days working before the same number of consecutive days off duty). The X axis is the length of working shift; Y axis is the attack rates.

Figure 4 shows the impact of different HCWs’ work shift length. There appears to be complex patterns, but within each testing strategy, the effects appear to be marginal, not exceeding 0.051 for any of the three testing strategies evaluated.

## Discussion

### Summary

Nursing home residents are older and typically frailer than the general population in high-density contact networks and thus potentially more susceptible to severe illness and mortality from emerging infectious diseases. Non-pharmaceutical interventions can be effective system-level countermeasures to mitigate the risk of severe illness and mortality. This research implements a realistic ABM simulation utilizing fine-grained spatiotemporal information from a sensor mote deployment in a nursing home. We use our simulations to evaluate the effect of four non-pharmaceutical interventions on reducing resident infection rates: (i) screening and isolation, (ii) inter-resident contact restrictions, (iii) reducing HCW presenteeism, and (iv) altering HCW scheduling. By considering all four interventions in the same modeling framework, it becomes easier to meaningfully compare individual or combinations of mitigation measures directly.

Overall, inter-resident contact restrictions were most efficacious, with regular screening and isolation making further substantial gains in reducing the attack rate. Reducing presenteeism was highly effective but only in the absence of regular screening and isolation. Regular screening and isolation alone were insufficient to control transmission; in contrast, restricting inter-resident contacts to bubbles of size four was quite effective alone, although not as effective as the combination of the two strategies.

### Effects of bubbling

While the contact patterns impacted the attack rate significantly, this difference depended on the community prevalence and the transmissibility of the disease. Doubling the size of each bubble from 4 to 8 only increased the attack rate by 0.06 for 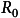 = 3.15 and community prevalence of 0.01, whereas this increase climbed up to 0.36 for 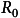 = 5.94 and prevalence of 0.1, suggesting stricter contact restrictions are necessary for highly transmissible diseases with high prevalence.

Fully eliminating contact between residents effectively reduced the attack rate, coming, however, at a very high cost to residents. In a scoping review, Rodrigues et al^23^ found that the social isolation of older adults during the COVID-19 pandemic increased loneliness, worry, stress, anxiety, fear, frustration, boredom, depression, sleep disorders, and suicide ideation. Bubbling can help lower infection rates while diminishing the negative psychosocial effects of isolating during an infectious disease outbreak.^24^ In our study, we found that on average the attack rate from the simulated outbreak was kept below 0.1 when bubbling groups of four residents together for up to 1.5 hours/day in all situations except for when the community prevalence was at 0.1 or when 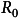 = 5.94 with the prevalence ≥0.025; bubbling up to 1 hour/day kept the attack rate below 0.1 unless 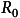 = 5.94 with the ≥0.05. These results suggest that bubbling can be highly effective at reducing transmission while potentially lowering the negative impacts of social isolation on nursing home residents’ mental health.

### Effects of presenteeism

The effect of presenteeism, a common issue with HCWs,^25^ was negated in the presence of regular screening. In the absence of screening, the attack rate was reduced from 0.49 to 0.31 by halving the presenteeism rate from 0.82 to 0.41. These results imply that management should prioritize presenteeism reduction only when there is no regular screening and isolation.

### Screening regularity

Our simulations revealed only small improvements when increasing screening rates from every 6 days to every other day. Moreover, the efficacy of screening was highly dependent upon the contact pattern of residents. With homogeneous contact patterns at 1 hour/day, the attack rates for testing every 6 days versus every other day were 0.41 and 0.20, respectively, while for bubbles of size 4, the attack rates were 0.03 and 0.02, respectively. However, in all cases, failing to screen and isolate positives led to much higher attack rates; with the prevalence of 0.025 and 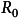 = 3.15, moving from no testing to testing and isolating every 6 days decreased the attack rate by 35%–79%, depending on the contact pattern and time per day in contact.

### Non-effect of work shift alterations

We had hypothesized that the disease characteristics (time to symptom onset, infectiousness profile, etc), testing schedules, and the interaction between the two may lead to different work schedules having an effect on the attack rate. What we observed was a complicated pattern that yielded marginal differences. This seems to imply that a strong knowledge of disease characteristics and transmission is required to know the effect of the work shift schedule on the attack rate, but that even with such knowledge, modifying shift schedules has little impact.

### Implications

Bubbling in groups of 4 alongside screening and isolation every 6 days was typically sufficient to maintain a low attack rate in the midst of an epidemic. For 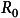 less than or equal to that of the wild strain SARS-CoV-2, if the community prevalence exceeds 0.05, the time bubbles spend together ought to be restricted to 1 hour per day. For larger 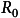 , when the prevalence exceeds 0.025, the time bubbles spend together ought to be restricted to 0.5 hour per day, and when the prevalence exceeds 0.05, inter-resident contact should be suspended until the community prevalence has reduced.

### Limitations

The results from this study should be interpreted in the light of several limitations. First, our ABM was derived from a sensor mote deployment in a single nursing home. This facility’s high-fidelity HCW location/interaction data may not apply to other facilities. Second, due to IRB considerations, we did not obtain patient locations, instead inferring their locations from HCW movements. Third, we focused on a respiratory disease characteristically similar to COVID-19, and our results may not generalize to other dissimilar diseases.

## Supporting information

Li et al. supplementary material Li et al. supplementary material

## Supplementary material

The supplementary material for this article can be found at https://doi.org/10.1017/ice.2024.43.
