# Supplementary material for: Comparing multiple infection control measures in a nursing home setting: a simulation study
Source: Infect Control Hosp Epidemiol. 2024 Mar 15;45(7):872–9. doi: 10.1017/ice.2024.43 (PMC11439596; doi:10.1017/ice.2024.43)
Supplement: Li et al. supplementary material [file S0899823X24000436sup001.docx]

**Supplemental Material for “Comparing multiple infection control measures in a nursing home setting: a simulation study”**

Haomin Li, M.S. Department of Biostatistics, University of Iowa, Iowa City, Iowa

Daniel K. Sewell*, Ph.D. Department of Biostatistics, University of Iowa, Iowa City, Iowa

Ted Herman, Ph.D. Department of Computer Science, University of Iowa, Iowa City, Iowa

Sriram V. Pemmeraju, Ph.D. Department of Computer Science, University of Iowa, Iowa City, Iowa

Alberto M. Segre, Ph.D. Department of Computer Science, University of Iowa, Iowa City, Iowa

Aaron C. Miller, Ph.D. Department of Internal Medicine, University of Iowa, Iowa City, Iowa

Philip M. Polgreen, M.D. Departments of Internal Medicine and Epidemiology, University of Iowa, Iowa

| **SM Figure 1.** Barplot of the number of healthcare workers (HCWs) each resident interacts with each day. On average, each resident contacted 7.8 HCWs, ranging from 1 to 15. |
| --- |
| 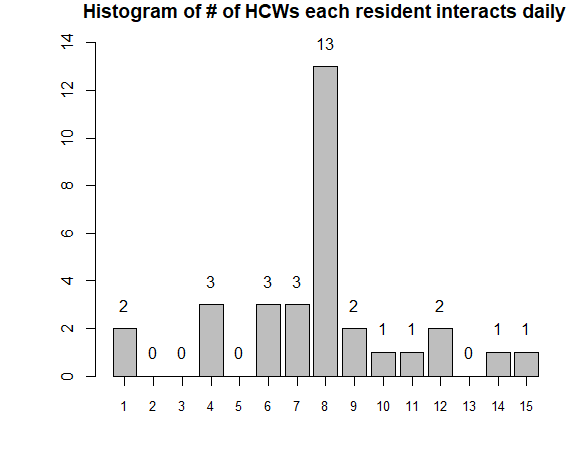 |
|  |

| **SM Figure 2.** Density plot of the time each resident spends in the same room with each HCW. On average, residents spend 36.7 minutes in the same room as another HCW, ranging from 1 minute to 3.5 hours. |
| --- |
| 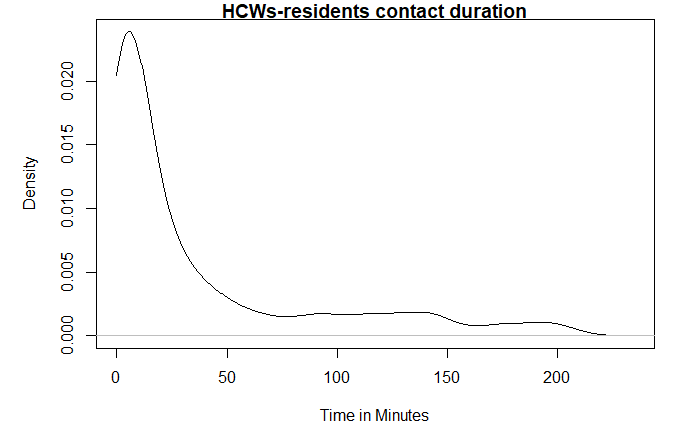 |

| **SM Figure 3.** Barplot of the number of residents each resident interacts with daily. On average, each resident was in contact with 4.7 residents, ranging from 0 to 15. |
| --- |
| 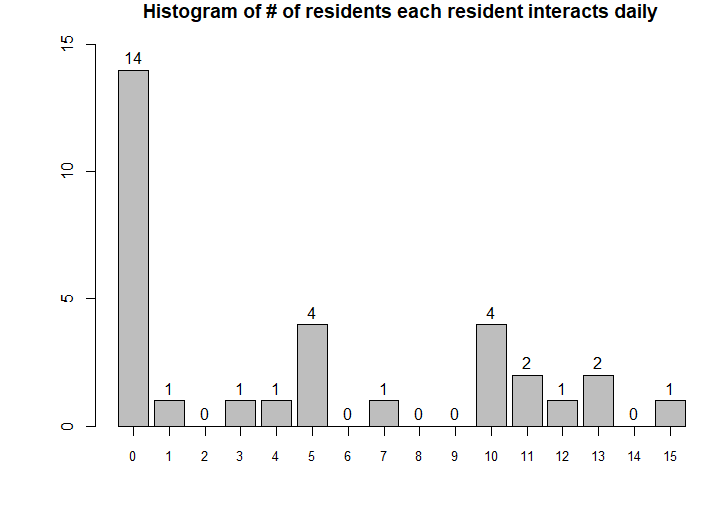 |

| **SM Figure 4.** Barplot of the daily number of HCW interactions for each HCW. On average, each HCW interacted with another HCW 10.7 times per day. |
| --- |
| 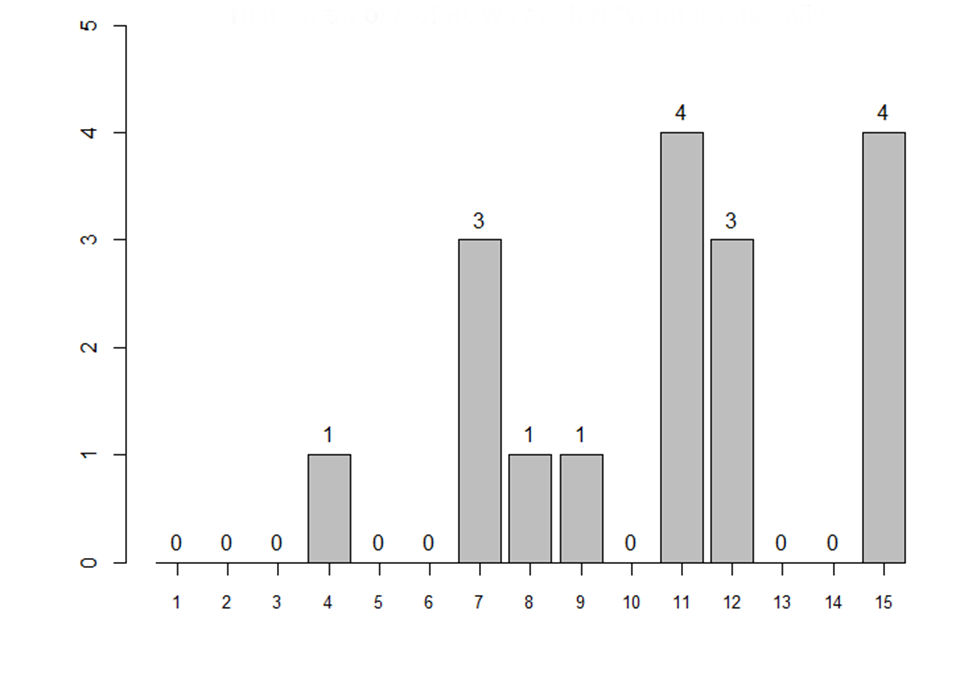 |

| **SM Figure 5.** Density plot of the length of time each pair of HCWs are in the same room. On average, each HCW-HCW interaction lasts 41.1 minutes, ranging from 1 minute to 4.4 hours. |
| --- |
| 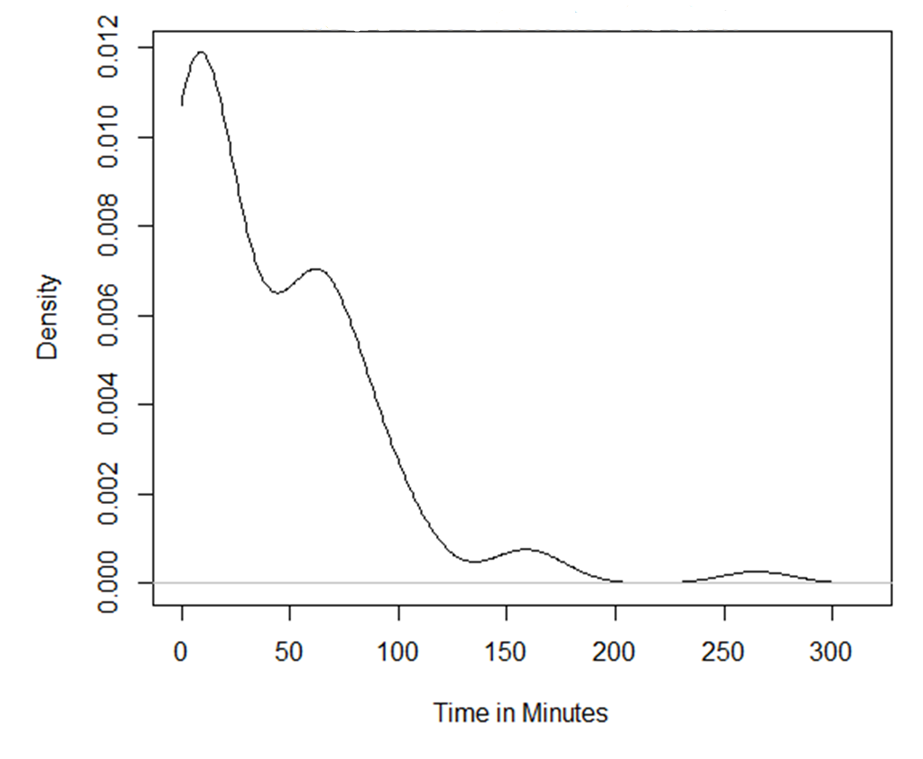 |

| **SM Figure 6.** Barplots showing the number of residents in each of the two common rooms in each 15 minute period. |
| --- |
| 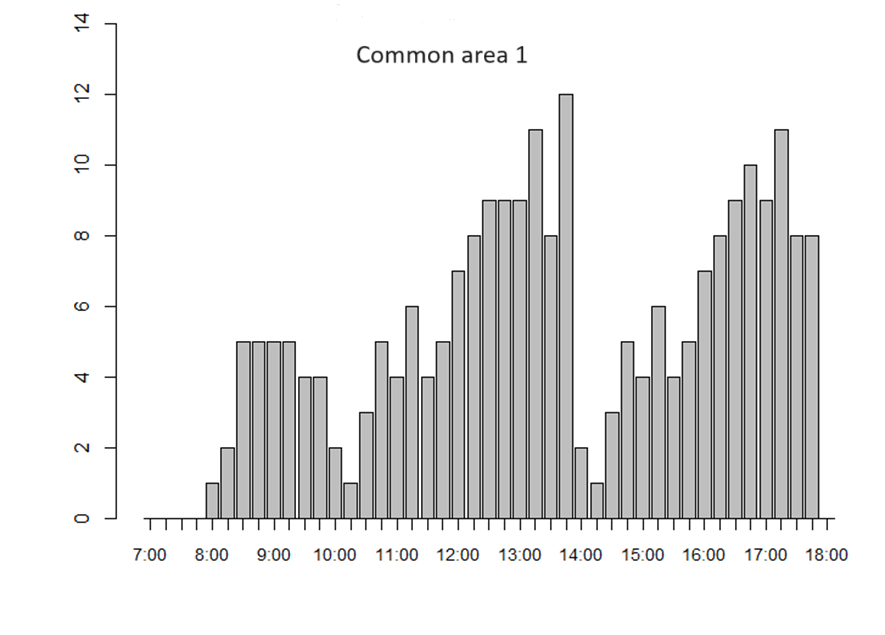  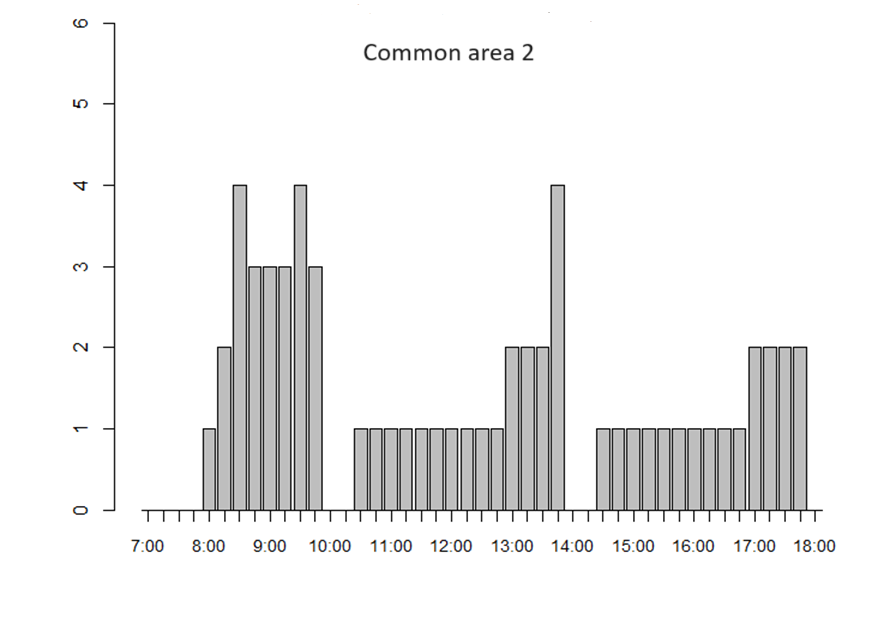 |

| **SM Figure 7.** Attack rates for HCWs. (A) residents contact each other homogeneously; (B) residents contact each other within 4 bubbles; (C) residents contact each other within 8 bubbles. |
| --- |
| 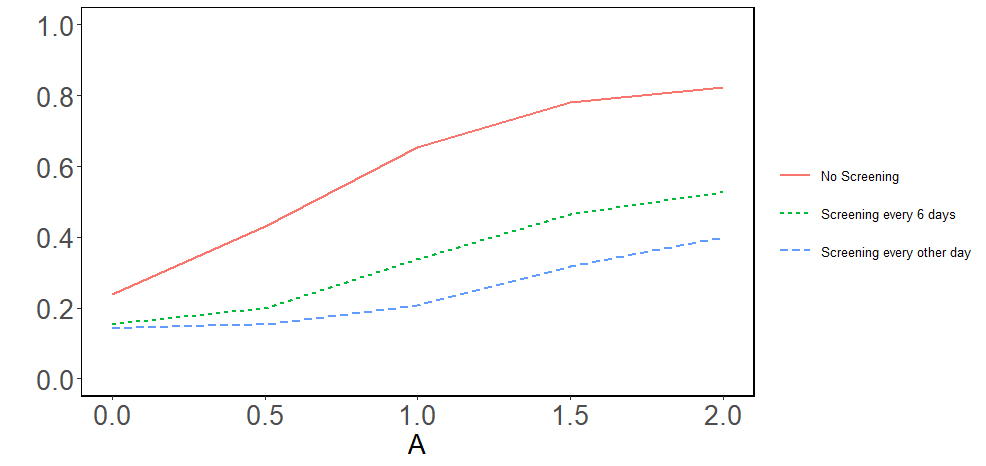  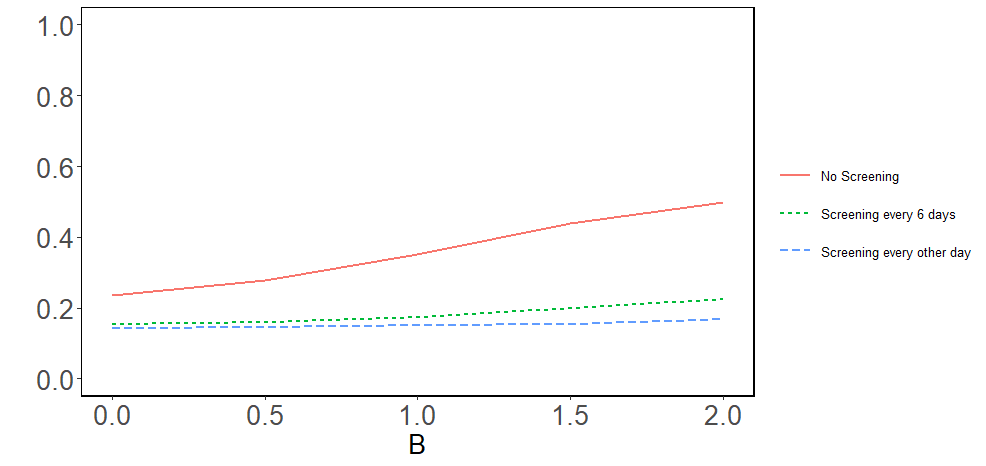  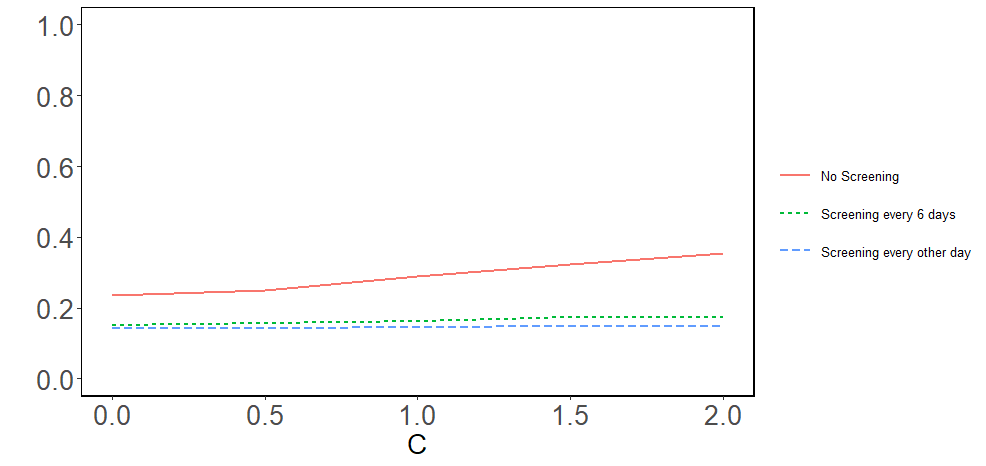 |

| **SM Figure 8.** Diagnostic sensitivity curve. Sensitivity is determined by the day relative to symptom onset. The red line is the raw data from Kucirka et al. (2020), and the black curve- used in our simulations- has been smoothed via spline regression. |
| --- |
| 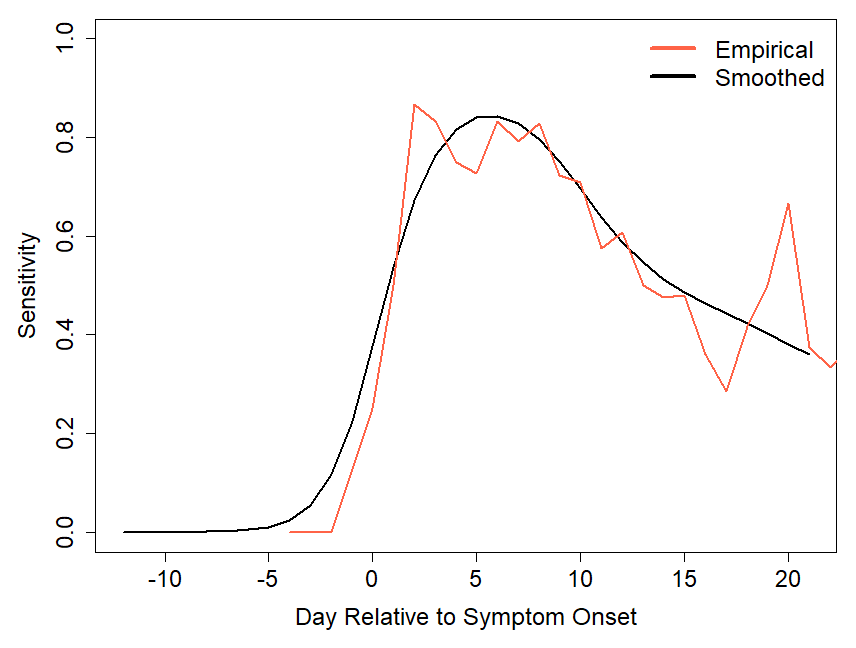 |
